# Review of Current Guided Wave Ultrasonic Testing (GWUT) Limitations and Future Directions

**DOI:** 10.3390/s21030811

**Published:** 2021-01-26

**Authors:** Samuel Chukwuemeka Olisa, Muhammad A. Khan, Andrew Starr

**Affiliations:** School of Transport, Aerospace and Manufacturing, Cranfield University, Bedford MK43 0AL, UK; s.c.olisa@cranfield.ac.uk (S.C.O.); a.starr@cranfield.ac.uk (A.S.)

**Keywords:** damage index (DI), ultrasonic guided wave (UGW), guided wave ultrasonic testing (GWUT), lamb wave, damage detection

## Abstract

Damage is an inevitable occurrence in metallic structures and when unchecked could result in a catastrophic breakdown of structural assets. Non-destructive evaluation (NDE) is adopted in industries for assessment and health inspection of structural assets. Prominent among the NDE techniques is guided wave ultrasonic testing (GWUT). This method is cost-effective and possesses an enormous capability for long-range inspection of corroded structures, detection of sundries of crack and other metallic damage structures at low frequency and energy attenuation. However, the parametric features of the GWUT are affected by structural and environmental operating conditions and result in masking damage signal. Most studies focused on identifying individual damage under varying conditions while combined damage phenomena can coexist in structure and hasten its deterioration. Hence, it is an impending task to study the effect of combined damage on a structure under varying conditions and correlate it with GWUT parametric features. In this respect, this work reviewed the literature on UGWs, damage inspection, severity, temperature influence on the guided wave and parametric characteristics of the inspecting wave. The review is limited to the piezoelectric transduction unit. It was keenly observed that no significant work had been done to correlate the parametric feature of GWUT with combined damage effect under varying conditions. It is therefore proposed to investigate this impending task.

## 1. Introduction

Metallic structures are essential in our society as they are used to perform crucial functions of economic importance. Some of these structures—such as oil pipelines, railway, aircraft, bridges, etc.—support or convey products prone to hazard. Structures are abounded to age and deteriorate due to changes in their material properties [1,2]. The properties could be mechanical—such as elastic Young’s modulus E, Poisson ratio ν, velocity v, density ρ—or geometrical properties—such as length, thickness, or shape [2]. Load stress and environmental operation conditions effect on the structures contributes to its property variation [3,4,5,6,7,8]. The accumulation of these changes over time results in a catastrophic structural failure that may be detrimental to the economy, environment and human lives [9]. Hence, appraising structure health state is imperative and necessitates approaches and techniques that could offer the needed information quickly, accurately, and reliably.

Conventionally, inspection for damage on a structure can be appraised visually and manually but principally limited to its surface access. There is a need for damage inspection beyond the surface, hence the non-destructive testing, NDT [10,11]. Researchers and industries adopt non-destructive testing to inspect the internal and external health condition of structures without destroying it [9]. NDT provides the capability to monitor the structures’ health state and detect flaws, thereby impeding and averting possible hazardous failures.

Prominent among these techniques is the guided wave ultrasonic testing (GWUT) method. It is an effective method for long-distance inspection of inaccessible and coated structures from a single point of testing. It propagates inspection wave all through the structure without point-to-point scanning, hence, minimizes inspection drudgery and enhances work throughput per day.

**Figure 1:**
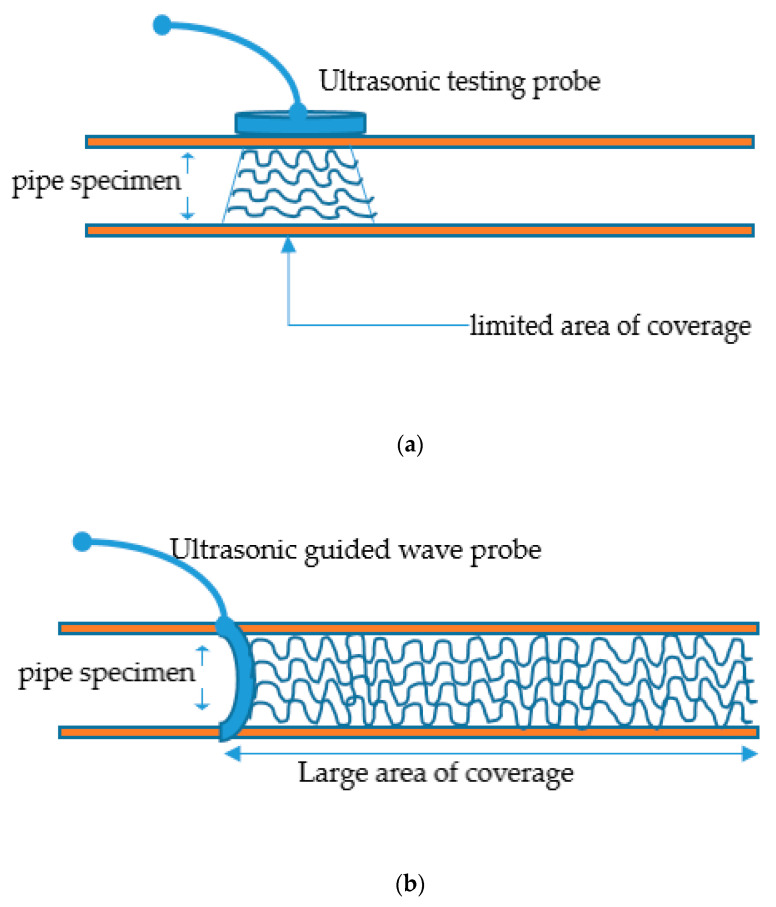
Difference between (**a**) ultrasonic testing, UT; and (**b**) guided wave ultrasonic testing, GWUT.

The conventional ultrasonic testing (UT) whose frequency is dependent on the thickness of the inspecting structure is different from GWUT of frequency range 20 kHz through 15 MHz or more in rare cases [12,13]. The UT is a high-frequency pulse-echo inspection technique for deep penetration into structural thickness [14]. It covers a limited area of the structure, unlike the GWUT that propagates a long-distance structure, as shown in Figure 1a,b.

In GWUT or long-range ultrasonic testing, LRUT, transducer probes are networked in an effective array manner to emit wave signals of predefined frequency [14]. The signal’s low-frequency nature allows the elastic wave to be guided and propagated a long distance at the expense of sensitivity. At the same time, inspection at high frequency is detrimental to distance coverage and power. The high-frequency wave signals are mostly used to study microscale changes in the material structure like corrosion. In contrast, low-frequency signals are majorly used to inspect and detect macroscale defects in the material structures. Most studies on the application of GWUT have focused on individual damage inspection under varying conditions. However, the phenomenon of coexistence of combined damage on structures and its effect on GWUT parametric features under varying conditions are yet to be studied. Hence, the review on GWUT damage inspection, severity, and temperature influences on the guided wave and parametric features of the inspecting wave.

## 2. Ultrasonic Guided Wave, UGW

In nature, dolphins and bats use echolocation technique to survive in their immediate habitats by processing an emitted and sensed signals [15,16,17,18]. They use the time of flight, ToF, of the signal to infer the object’s location ahead, thereby manipulating their travelling intention [19]. Their call echo signal’s parametric features have been extensively studied as a tool through which they differentiate fishes and detect hidden prey in sediments [20,21,22,23]. This approach is similarly adopted and extensively researched to inspect and monitor structures’ health state using parametric features of ultrasonic wave and the guided wave geometry [24,25,26]. As sound wave propagation is stratified in different frequency ranges, an ultrasonic wave of about 20 kHz–25 MHz is widely used for monitoring and screening of structural assets [24,27,28,29,30,31,32]. The screening and monitoring at less than 100 KHz is regarded as low frequency [29,33], which is predominantly used in long-range screening of about 80–100 km while above 100 kHz is considered as high-frequency screening [34,35]. The propagating ultrasonic wave interaction with the material boundaries and surrounding media affects its parametric features and wave modes, especially when encountered discontinuity, flaws, notch, and corrosion [34]. The interaction of the propagating wave with structural damage results in different effects such as wave absorption, scattering, attenuation, and reflection [10,36,37]. These effects are often proportional to the severity of the encountered defect in the structures [30]. The increase in the number of sensors increases the chances of receiving all scattered damage signals for accurate location and quantification of the damage but not cost effective [14].

A defect in structure is indispensable as it could occur at the manufacturing stage or during service life through dynamic loading, ageing, environmental effects, and corrosion activities [38,39]. All these factors affect the strength of structure through variation in its flexural rigidity or mass that manifests in vibrational frequency [18,40,41,42,43] of the structure as in Equation (1).

(1) $$f_{i}=\frac{\lambda_{i}^{2}}{2\pi L^{2}}{\left(\frac{EI}{m}\right)}^{1/2}$$

where $f_{i}$ is the linear natural frequency,$\;\lambda_{i}$ is modal constant, L is the beam length, EI is the flexural rigidity of the beam while m is the mass. The deterioration of assets if not detected, quantified, localized, and an appropriate maintenance measure administered on time could result in catastrophic failure that might claim lives, harm the environment and cripple the economy. Hence, predicting the inception of the damage existence early before it becomes severe is eminent. However, at the initial stage of the flaw, its size could be microscale and hard to detect, especially in a coated scenario.

In asset integrity assessment, damage identification is of four spectrums [41]:(i) damage detection, (ii) damage localization, (iii) damage quantification, and (iv) damage prognosis. Some studies emphasized on one or any of the combinations. However, the limitation could be attributed to the assessment technique employed as some could only provide information for the awareness and location of the defect.

Integrity assessment techniques used in determining the health of structures could be passive or active when referenced to the actuation nature of the structure [19,27,44,45,46,47,48,49,50,51]. The essence of actuation is to set the structure particles into mechanical vibration that would result in momentary spatial displacement. In passive actuation method, ultrasonic sensors are sparsely deployed to sense the structure health status when it is under dynamic stress that results in modal shape deformation and vibrations, thereby emitting a sound of different frequencies. Instances of this technique are the strategic placement of acoustic emission (AE) [19] sensor and fiber Bragg grating (FBG) [52] for sensing the variations in the structure sound frequency and strain caused by the existence of defect respectively. FBG fundamentally operates in the principle of reflection and filtering light signature wavelength whose variation is proportional to the structural strain [52] as in Equation (2).

(2) $$\frac{\Delta \lambda_{B}}{\lambda_{B}}=\left\{1-\frac{\eta_{e}^{2}}{2}\left[p_{12}-\nu \left(p_{12}+p_{12}\right)\right]\right\}$$

where $\lambda_{B}$ is Bragg wavelength, $\eta_{e}$ is the Bragg effective refractive index, $p_{ij}$ are the silica-photo-elastic tensor components, ν is the Poisson’s ratio, and ε is the strain.

Although passive actuation method overcame some of the challenges of the conventional ultrasonic sensor in terms of increased sensitivity, shield to electrical noise and durability, the sparse placement of transducers on structure creates the possibility of flaw detection miss. This challenge could be adequately handled by increasing the number of sensor per area but at the expense of cost.

On the other hand, the active actuation method works with at least two transducers (lead zirconate titanate, PZT) to act as an actuator and as a sensor synchronously without recourse load-induced sound wave occurred in the structure [19]. Hence, they are nonresonant sensors unlike passive actuation method and could be networked in phase array or distributed network that offers full coverage of the inspecting structure with possible wireless data communication interface [19,24]. The lightweight nature, small size, easy integration of PZT on host structure coupled with cost-effectiveness made it an excellent choice for structural health monitoring [26,53], especially for thin plate structures and composite materials that are predominantly used in aircraft constructions. Prominent industries of NDT have used it to develop PZT arrays of various inspection specifications of 15–100 KHz frequency range and 60–150 m inspection distance [54,55,56,57].

Lamb wave is prominent for the excitation of GWUT in thin plates and other flat composite structures because it influences structure properties and propagates useful signal information regarding the structure’s integrity. It is sensitive to sundry defects and discontinuity that manifests in its in-plane S_0_ and out-plane A_0_ modes [58,59,60,61,62]. These are fundamental modes of symmetric and antisymmetric modes of Lamb wave that are dispersive. The propagation speed of symmetric, S_i_ modes is faster than antisymmetric A_i_ modes [62]. The frequency of the guided wave and thickness of the inspecting structure correlatively defines the lamb wave features’ varying nature. Hence, the product of the frequency, f and the structural thickness, d (f–d) relate proportionally to the Lamb wave modes [63]. It is pertinent to point out that fundamental lamb wave modes exist majorly at smaller product value of f–d. The antisymmetric modes have been revealed to be more sensitive to defects at a higher frequency than the symmetric mode in detecting microcracks [63,64,65]. It was revealed in the study done by Alleyne and Cawley in [64] that the sensitivity of the fundamental modes of the lamb waves are functions of damage depth, *h* to guided wave thickness, *d* ratio ($\frac{h}{2d}$).

In a plate–water interface, the in-plane wave mode, S_0_ remains the same while partly energy of the out-plane wave mode, A_0_ leaks into the water. This is an attribute to differentiate media acoustic properties [66]. Contrary to this, both modes attribute to leaky wave in the case of plate–soil interface. Although lamb wave propagates at high velocity, it is susceptible to the effects of ambient noise, low-structural vibration and temperature, which harden the ease of damage identification and interpretations [67]. In pipe diagnosis and at low frequency, the longitudinal, L; torsional, T; and flexural waves, F, are dominant [24,28,68,69]. Aside from the fundamental mechanical waves, other waves formation are feasible but derivate of them [12,31,70] and occurs in different structural conditions. To appreciably differentiate one wave mode from the other, a naming nomenclature was devised by Meitzler in [71] as in Equation (3).

(3) Wave modes = X(h,g),

The *X* represents the wave mode type (Longitudinal, L; Torsional, T, or flexural, F) while the h and g are circumferential order and mode order, respectively. The circumferential order and wave mode order of the longitudinal and torsional waves are similar in convention and differ from the flexural mode. This often occurs due to wave mode conversion of either the longitudinal or torsional modes [72].

### 2.1. Wave Propagation and Dispersion

Since wave propagation brings about vibration and displacement of structural particles in the temporal and spatial directions, the fusion of the Hooke’s law and Newton’s law results in a 1D governing wave equation [71] Equation (4).

(4) $$\frac{\partial^{2}u}{\partial x^{2}}=\frac{\rho_{0}}{c}\frac{\partial^{2}u}{\partial t^{2}}$$

where u is the particle displacement, x is the propagation direction, $\rho_{0}$ is the density of the unstressed structure, c is the elastic constant of the structure, and t is the time. Furthermore, the resolution of the harmonic propagating waves of different frequencies but equal amplitude results in two-term frequencies as in Equation (5).

(5) $$u=2A\cos\left(\frac{1}{2}\Delta kx-\frac{1}{2}\Delta \omega t\right)*\cos\left(kx-\omega t\right)$$

The first term of (5) is of low frequency with limit group velocity as in Equation (6).

(6) $$C_{g}=\frac{\partial \omega }{\partial k}$$

While the second term is of high frequency with phase velocity as in Equation (7)

(7) $$C_{p}=\frac{\omega }{k}$$

By comparing Equations (6) and (7), it implies that group velocity and phase velocity are related as in Equation (8).

(8) $$C_{g}=\frac{d\left(kC_{p}\right)}{dk}=\;C_{p}+k\frac{dc_{p}}{dk}$$

where $C_{p}$ = $C_{p}\left(k\right)$.

It is pertinent to note that this wave propagation in lossless elastic structure is characterized by group velocity, $C_{g}$ and phase velocity, $C_{p}$. The group velocity is a function of the central frequency, f, and the thickness, d of the guided wave as in Equation (9).

(9) $$C_{g}\left(f.d\right)=\frac{C_{p}^{2}}{\left[C_{p}-\left(f.d\right)\frac{dc_{p}}{d\left(f.d\right)}\right]}$$

Equally, selecting the distinct wave mode for inspection is substantial for accurate quantitative and qualitative damage detection [73]. However, it is a challenge to achieve this due to the lamb wave’s multi-mode nature at different frequencies when the inspection wave mode is supposed to be nondispersive [25]. Hence, in 1990, Mike Lowe and Brian Pavlakovic of Imperial college London developed structure mode evaluation software called ‘Disperse’ [74]. It predicts possible modes and characteristics of a wave propagating in a structure [75,76]. This software is prominent and extensively used in NDE and NDT of structures.

**Figure 2:**
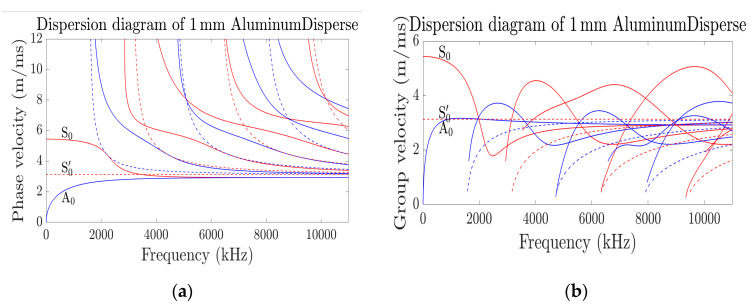
(**a**,**b**) Dispersive curve of 1 mm thickness of Al obtained using DC [77].

Similarly, in 2016 Armin Huber of the Lightweight Production Technology of the German Aerospace Center developed ‘Dispersion Calculator’ (DC) [75,77]. DC is more intuitive and interacting than the famous disperse software [76,77]. The calculator uses the stiffness matrix method formulated by S. I. Rokhlin and L. Wang [78,79,80] to compute the dispersion curve of longitudinal and shear waves in an anisotropic plate and multilayered transversely isotropic composites. Figure 2 is the guided wave phase velocity, and group velocity dispersive curves of 1 mm aluminum plate thickness generated using the DC. It could be deduced from the dispersive curve of phase velocity that $S_{0}$ mode is relatively nondispersive up to 200 kHz with $C_{p}$ of about 5430 m/s when compared with other modes. Therefore, it is suitable to monitor the health condition of the plate using $S_{0}$ mode as the generated excitation signal. However, excitability and sensitivity of the modes, especially with regards to thickness reduction due to corrosion was not accounted for. In [73], an optimized dispersion curve that relies on the excitability and sensitivity in addition to the group velocity of the mode was developed. This was named goodness dispersion curve, and it offers an informed mode suitable for damage detection but limited to material thickness reduction due to general corrosion. It was revealed that sensitivity of guided wave varies with wave frequency and at low frequency, the wave is insensitive to small thickness variation of the metal. Interestingly, the fundamental symmetric, S_0_, and asymmetric, A_0_, modes were recorded as insensitive.

### 2.2. Transducer Arrangement and Configuration for Inspection of Structure

Structure inspection involves the use of transducers as an actuator (exciter or emitter of pressure waves) and a sensor (receiver or catcher of pressure wave) to generate and receive wave through a waveguide at a predefined distance. The transducer’s major component is the piezoelectric material that can transform electrical energy into mechanical vibrations and vice versa [81,82]. The relationship between the material’s electrical and mechanical effects is in Equations (10) and (11). The parameters of the equations are defined in Table 1.

(10) $$T_{j}=C_{ji}^{E}S_{i}-e_{jj}E_{j}$$

(11) $$D_{i}=\epsilon_{ij}^{s}E_{j}+e_{ii}S_{i}$$

**Figure 3:**
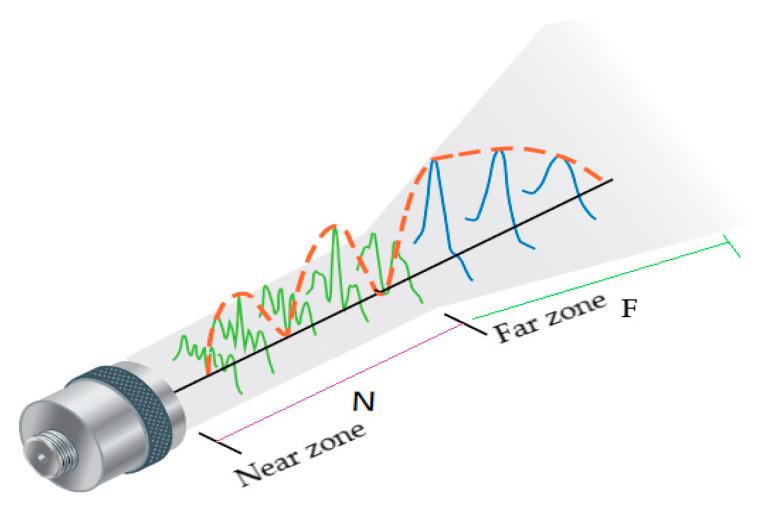
Transducer wave zones [84].

The transducer pressure wave is of two-zone formation [83]: the near zone and far zone, as shown in Figure 3. At the near zone, the wave pressure amplitude varies spuriously with many high and low echoes. However, beyond the near zone distance *N*, the wave gradually attenuates in the amplitude. The multiple echoes at the near zone make it challenging to detect structural damage between the near zone and transducer. This is a blind spot of the transducer where damage detection through signal amplitude analysis is ineffective. Hence, damage detection is effective beyond the near zone distance and within the far zone. The near zone distance is defined as in Equation (12). D is the transducer diameter, f is the pressure wave frequency, λ is the wavelength, v is the velocity.

(12) $$N=\frac{D^{2}f}{4v}$$

The effectiveness of structural inspection in one hand relies on the damping nature and its spectral response to the targeted damage. On this account, transducers are grouped into three and summarized in Table 2.

**Figure 4:**
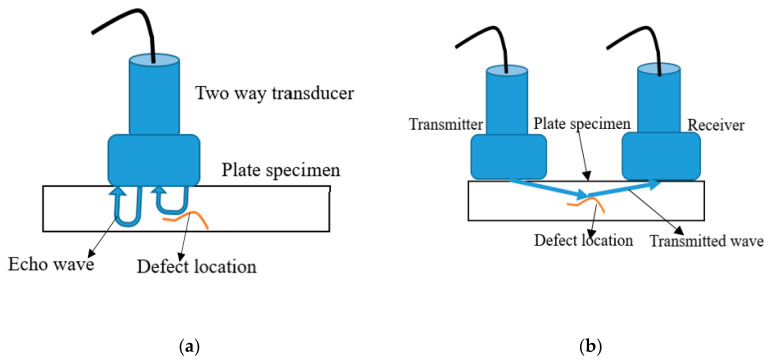
(**a**) Pulse–-echo method with a transducer; (**b**) Pitch–catch method with two t ransducers(R) [86].

The configuration topology of the transducer is majorly either pulse-echo (P–E) or pitch-catch (P–C) depending on the geometry of the inspecting structure, targeted defect of interest and the needed data [34]. The typical examples of the methods are shown in Figure 4. In P–E, a single or two transducer(s) is used, and the reflected echo signal, time-of-flight (ToF) alongside the propagation velocity are used to determine defect, deterioration, discontinuity, or thickness of the material structure [70,85]. A minimum of two transducers is required in P–C and spaced sufficiently to generate defect interrogating wave and receive it after interrogation with the structural defect. This configuration is often applied to detect or monitor defects that are not sufficient to cause reflection somewhat further transmission of the wave though attenuated. Unlike the P–E method, the P–C measurement is the amplitude of the damage signal or the received damage signal’s shape. P–C is an excellent choice to make when the intention is to cover a long-range structure [86].

**Figure 5:**
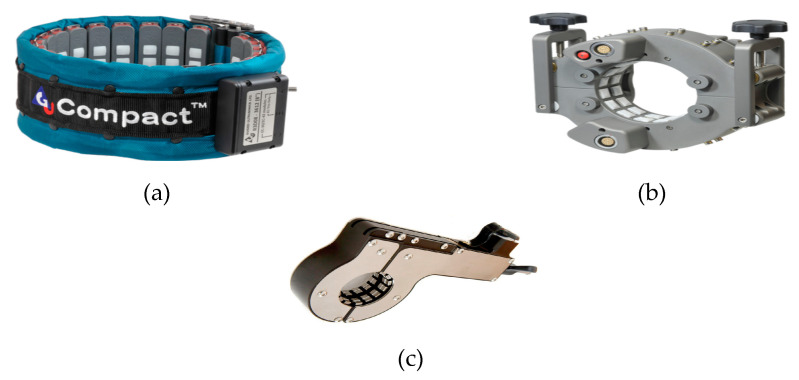
(**a**) Compact ring transducer; (**b**) Solid ring transducer; (**c**) Claw transducer [87].

In pipeline inspection, piezoelectric transducers are distributed and interconnected in parallel to form a ring of an array around the pipeline’s circumference [44,45,46]. This technique of inspecting the pipeline is devoid of surface preparation unlike other types of inspecting technique; hence, saves time and encourages more length inspection in a day. However, hopping the device from one point to another is laborious, coupled with its weight, installation, and uninstallation time at distances of interest. Some of the available collar ring transducers in the market for a specific inspection challenge are shown in Figure 5. The compact ring is designed for a wide range of inspection with a 30–35% weight reduction. The solid ring transducer type is for standard inspection of 2–8 inches pipes while the claw transducer type is designed to inspect a closely spaced pipeline that cannot allow the fitting of the conventional ring [87]. A typical transducer ring array contains 2–24 transducers depending on the diameter of the pipe [28,88,89].

**Figure 6:**
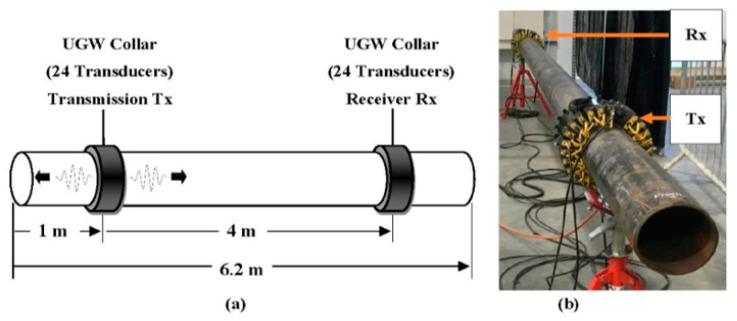
(**a**) Schematic and (**b**) setting-up of the UGW collar arrangement for pitch–catch configuration topology [89].

Therefore, about 48 transducers must form two rings of circumferential inspection array of a sizeable guided wave. The high number of transducers per ring adds to the high cost of inspecting structures. The networking together of such a large number of transducers could pose a challenge in determining when a transducer has failed. Although it offers merit in full inspection coverage of the structure, it increases the system inspection power [71]. Also, the large size of the collar transducers does not encourage in-service inspection of structure which demands permanent installation of the device [90]. In Figure 6, two 24-transducers collars were configured in pitch-catch topology and used to inspect internal corrosion activities in a pipeline [89]. In [28], multiple ring arrays of 24 transducers were used to study the guided wave’s unidirectional excitation function. Fundamental Torsional (0,1) wave mode was generated and conditioned to propagate only in a forward direction. This approach reduces complexity in the signal collection and interpretation, and it saves computational cost.

### 2.3. Attenuation of Propagating Wave

**Figure 7:**
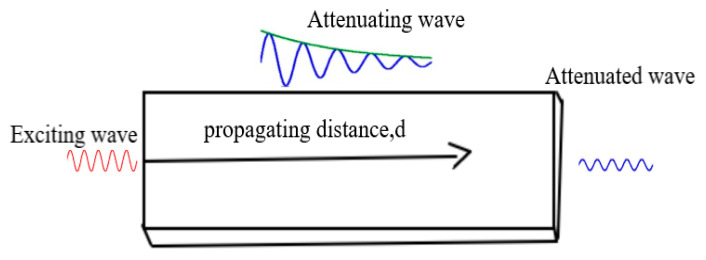
Signal attenuation in a medium.

Attenuation reduces signal level, either in amplitude or energy as it propagates through a medium [81]. Figure 7 is a typical demonstration of wave attenuation in a medium. The diminishing of the propagating signal amplitude often follows an exponential decay pattern expressed in Equation (13) while variation in the signal intensity is described in Equation (14).

(13) $$A=A_{0}e^{-\sigma x}$$

where *A* = Attenuated signal amplitude

$A_{0}$ = unattenuated signal amplitude
σ = coeffiecient of attenuation
x = signal travelled distance

(14) $$\Delta I\left(dB\right)=10\log\frac{P_{2}^{2}}{P_{1}^{2}}=20\log\frac{V_{2}}{V_{1}}$$

where ΔI = variation is sound intensity between two successive measurements, $p_{1}$ and $p_{2}$ are successive sound pressure, $V_{1}$ and $V_{2}$ are successive output voltage of the transducer. In [91], ultrasonic wave attenuation was statistically correlated with the strength of low carbon steel. The study showed that as wave attenuation increases from 0.24–0.36 dB/mm, the steel strength decreases from 290–460 MPa. This is a linear relationship suggesting that a structure’s strength can be monitored through a propagating wave’s attenuation level. The wave attenuation α and material thickness, *X* are related as in Equation (15). ΔA is the change in wave amplitude between successive reflections.

(15) $$\alpha =\;\frac{20}{2X}\log\left(\Delta A\right)$$

Aside from reflection, wave dispersion and waveform alteration, the combined effects of wave signal scattering and absorption results in signal attenuation. Hence, attenuation is a significant effect that happens to wave propagating in structures.

As expected in reality, the propagating wave will attenuate over a distance of the guided medium. This could be due to losses resulting from leaking wave energy from the monitoring structure, interaction with the corroded area, defect, flange, welded area, and cracking. In NDT, attenuation is a linear effect that commensurates with the degree of defect in the material structure [30]. Accordingly, signal-to-noise ratio, SNR, is used to determine the level of attenuation undergone by a signal using input and output energies or amplitudes of the input and output wave strengths [69]. The GWUT is primarily an inspection/screening tool; hence SNR is suitable for determining the activities of an internal and external defect in the monitored structure. Environmental operating conditions also contribute to attenuation and distortion of damage signal information [92], thereby leading to false signal alarm, masking damage signal, and difficulty in defect localization. Temperature is a major environmental factor that causes damage signal distortion and diminishing, although scholarly works have been done to resolve its influence. It is pertinent to note that GWUT serves only the role of inspection and its capability to determine the size, shape, and orientation of crack has not been extensively studied [93,94,95]. In fluid-filled pipe inspection, the wave attenuation is affected by similarity and dissimilarity between the filled-fluid and the surrounding fluid [90]. It was revealed that, irrespective of the position of fixed structure features (weld, flange, etc.), attenuation rate in dissimilarity scenario is twice that of similarity. In a pipeline, attenuation is considered intrinsic when caused by absorption and extrinsic when caused by pipeline features like corrosions, cracks, and other defects that could decrease signal strength [90].

### 2.4. Damage Severity Indicator, DSI

Crucial in structural health monitoring is being aware of the damage present, especially at an early stage. This allows for an appropriate maintenance scheme to be applied on time and avoid unscheduled system shutdown due to structural damage. Several works have used lamb wave features extracted before and after the occurrence of damage or between two states condition of the structure to achieve this purpose [45,96,97]. The lamb wave feature extraction could be processed through an already established model or deterministic approach. The quantification of the lamb wave feature’s extracted damage information in any of the methods is the damage index (DI). The DI defines the severity of the damage in the structure and its health status. It could be classified as time-domain (root mean square (RMS), time of flight (ToF), wave energy attenuation, peak signal amplitude variation, and beat length), frequency-domain (figure of merit (FoM), spectral density, peak of FFT) or time-frequency domain as in Table 3. In [98], ToF was successfully used in the two comparable techniques to locate crack and its propagation in an aluminium plate. The continuous wavelet transform coefficient was used in [99] to detect thickness reduction in a thin aluminium plate.

In contrast, the amplitude coefficient peak was used in [58] to assess fatigue crack in an aluminium pipe. In [27], the beat wavelength was used to monitor thickness reduction due to generalized corrosion activities in the steel structure. In [45,100], different statistical damage indices (i.e., RMSD (Root-Mean-Square Deviation), MAPD (Mean-Absolute-Percentage Deviation), Cov (Covariance), TMAC (Transmissiblity Modal Assurance Criterion), and CC (Correlation Coefficient.)) were compared using PZT impedance measured under the uniformly corroded pipeline.

**Table 3:** Classification of DI according to domain.

Reference	DI Time-Based	DI Frequency-Based	Remark
[99]	Coefficient of CWT, *Z* = $\frac{\left(\frac{A}{B}+\frac{A}{C}\right)}{2}$ Where *A* = Fraction of the total energy of the CWT that lies at the centred frequency *B* = Fraction of the total energy of the CWT that lies at the higher frequency *C* = Fraction of the total energy of the CWT that lies at the lower frequency		Thickness reduction in a thin plate
[58]	Peak amplitude coefficient, $\beta =\frac{A_{2}}{A_{1}^{2}}$ Where *A*_1_ = amplitude of the fundamental wave and *A*_2_ = amplitude of the second harmonic		Fatigue crack propagation in aluminium pipe
[27]	The beat wavelength, $L=\frac{2\pi }{K_{A0}-K_{S0}}$ *K_A_*_0_ and *K_s_*_0_ are the wavenumbers of the fundamental symmetric and antisymmetric		Monitored thickness reduction due to general corrosion activity
[90]	$R_{ref}=\frac{A_{wel}}{A_{dir}}$ *A*_wel_ = Peak amplitude at the weld *A*_wel_ = Peak amplitude from a direct source		The severity of damage in the fluid-filled pipe
[101]		$DI=\left|1-\frac{fd^{T}fd}{fb^{T}fbL}\right|$ *fd* = the spectral signal frequency response at damage state *fbL* = the spectral signal frequency response at the undamaged state	Corrosion severity detection in pipeline
[59]		$DI_{ij}\left(f_{ex}\right)=\sum_{t}\left|V_{ij}^{B}\left(t,f_{ex}\right)-V_{ij}^{D}\left(t,f_{ex}\right)\right|$ for *i,j* = 1~6 where: *DI_ij_*(*f_ex_*) = Damage signal differential $V_{ij}^{B}\left(f_{ex}\right)$ = Baseline signal when pairing the *i*-th PZT actuator and the *j*-th PZT sensor at a given excitation frequency (*f_ex_*). $V_{ij}^{D}\left(f_{ex}\right)$ = damage signals when the corrosion damage was present at the targeted position of the plate.	Corrosion detection and severity in the plate Aluminium
[61]		Spectral density, $DI_{2}=\frac{\left|A\left(2\omega \right)\right|}{\left|A\left(\omega \right)\right|}$ Where|A(2ω)| = spectral magnitude of the fundamental frequency |A(ω)| = Spectral magnitude of the second harmonic frequency	Microscale crack detection in a plate structure

### 2.5. Corrosion and Sensitivity

**Figure 8:**
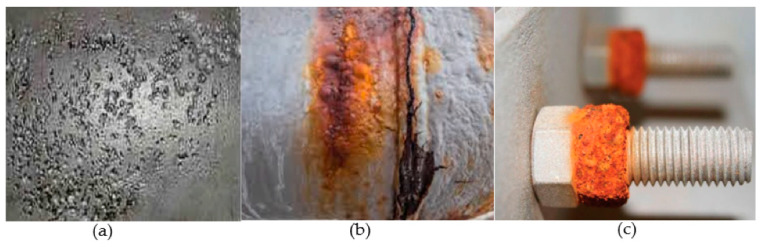
(**a**) Pitting corrosion in carbon steel [109]; (**b**) stress crack corrosion on carbon steel [110]; (**c**) crevice corrosion on a bolt [111].

Corrosion activity is an inevitable effect on metallic structures. It is one of the significant issues of safety in industries and results in corrosion failure of structures. It is instigated mostly through metal interaction with immediate environmental variables (oxygen in the air, hydrogen, chlorine, ambient temperature, humidity, and presence of microbial) or stress on the metallic structure as in the case of stress corrosion cracking [102,103,104]. The result of corrosion activity would be reddish rust if it happened in the presence of oxygen or greenish rust in the chlorine-dominated environment [105,106]. Some corrosion forms are pitting, crevice, and stress corrosion cracking with their peculiarities [107,108] as shown in Figure 8.

The pitting corrosion is caused by unevenness in the metal structure or breakdown of the metal surface’s protective coating, thereby exposing it to electron loss. It is not easy to predict and detect. Hence, it has the capability of causing corrosion failure and remain undetected. Crevice corrosion occurs due to an imbalance in ion concentration between the activity areas of the metal structure. It could happen in an oxygen-limited area such as under washers or bolts [111]. The growth of crack on ductile metal due to corrosive surroundings and applied cyclic stress is referred to as stress corrosion cracking (SCC) [112]. Corrosion rate on the material is estimated using Faraday’s law [113] of metal’s electrochemical reaction as in Equation (16).

(16) $$Corrosion\;rate\;\left(\frac{mm}{year}\right)=\frac{3.16\times 10^{8}\times i_{corr}\times M}{z\times F\times \rho }$$

where $i_{corr}$ is corrosion current, M is molar mass of the metal, z is the number of electrons transferred per metal atom, *F* is Faraday’s constant, and ρ is the metal density.

Corrosion can occur on plate and pipes but with different quantification approaches. Unlike plate, pipes’ defect size is quantified by the loss of wall area or thinning of pipe thickness [108]. This loss of wall area is termed percentage reduction in cross-sectional area, CSA, of pipe. This is on the account that propagating incident waves do not extend across the entire plate. Monitoring the corrosion activities and where it happens on the structure under different situations is of utmost importance to industries. Detecting corrosion activity of the least percentage of CSAL is a step in the early prevention of asset failure. This is plausible using a highly sensitive tool whose parameters can respond quickly to changes in the structure. The aftermath effect of corrosion activity is the deterioration of structural integrity through rust. The formation of rust involves loose surface properties of the structure, contamination of fluid in pipes, decreased service time, and a sudden failure of the structure that could cause loss of lives, and highly valued properties.

**Figure 9:**
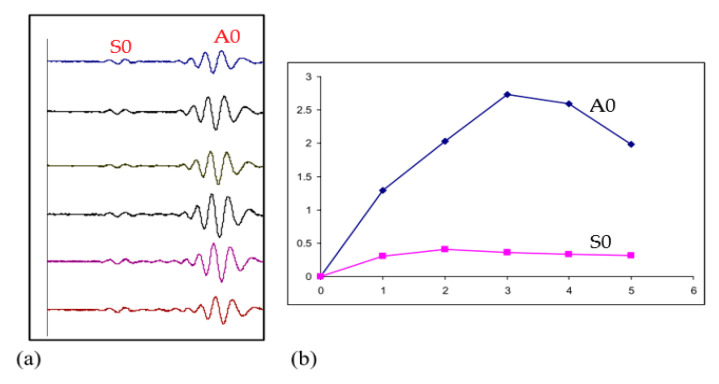
DI analysis on pitch-catch signals for corrosion detection. (**a**) Received signal at PWAS 4 when PWAS 0 sent at 120 kHz; (**b**) DI curves of both A0 and S0 wave packets at 120 kHz of path 0–4 [117].

High-frequency GWUT has been extensively used in monitoring corrosion activity in structures [63,114,115] although low-frequency monitoring was demonstrated in [116] using 64 PZT sensors. In [117], Victor Giurgiutiu et al. leveraged on piezoelectric wafer active sensor (PWAS’s) cheap cost, and networked multiples of it for corrosion detection and localization in plates and pipelines. Fundamental lamb wave modes S_0_ and A_0_ at high frequency (120 KHz) were generated and used. It was revealed that A_0_ is more sensitive than S_0_ in detecting corrosion both in plate and pipe although S_0_ travels faster but less sensitive to the metal thickness changes. This is depicted in Figure 9.

In [72], scattering and attenuation of propagating torsional wave in the pipeline with general corrosion were numerically and experimentally investigated using pulse-echo transducer configuration. In the study, rough surface mean depth and surface roughness were unified as corrosion activity. It was revealed that wave scattering is proportional to the unified corrosion activity. It implies that wave scattering increases as the pipe wall thickness reduction and rough surface area increases. This interaction effect significantly contributes to the wave attenuation and mode conversion from torsional T (0,1) wave mode to a higher flexural wave mode. Hence, corrosion development on structures impinges the propagating wave’s strength and promotes wave mode conversion that introduces background noise into the monitoring system.

**Figure 10:**
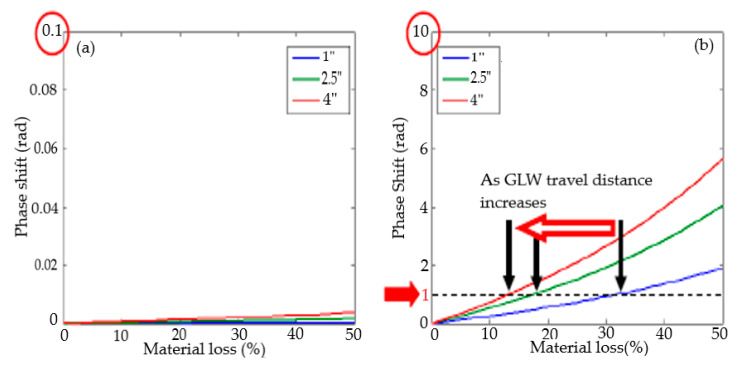
Phase shift estimation according to three-wave propagation distance (60 kHz excitation) (**a**) S0 mode (**b**) A0 mode of GLWAdpted with permission from [59]. Copyright 2012, ASME.

In [118], accelerated corrosion activity on a steel bar in concrete was successfully monitored using high frequency (200 KHz) lamb wave. The study revealed that corrosion in this nature could be detected using the FFT signal’s amplitude response. In [59], corrosion activity was experimentally monitored and quantified using a damage index profile map and A_0_ phase shift of the sensor signal. The A_0_ group velocity was observed to vary accordingly with the material loss from 0–50% of the thickness. The obtained result suggested that the phase shift of the fundamental antisymmetrical lamb wave A_0_ mode can be used to quantify early corrosion activity in structures, as shown in Figure 10.

In [116], real-time low-frequency (30–90 kHz) monitoring of corrosion on mild steel was studied. Fundamental antisymmetric wave mode, A_0_ was generated using 64 PZTs. It was revealed that the scattering waves’ influence on the accuracy of the thickness reconstruction is minimal. However, the thermal stability of the design on guided wave techniques was not considered.

In [101], PWAS configured in the pitch-catch mode were installed permanently on a SMART layer to perform in-service monitoring of the pipeline integrity. The severity of corrosion activity is determined via the damage index defined in Equation (17).

(17) $$DI=\left|1-\frac{fd^{T}fd}{fbL^{T}fbL}\right|$$

where

*DI* = the damage index for each affected paths
*fd* = the spectral signal frequency response at damage state
*fbL* = the spectral signal frequency response at the undamaged state

In [27], thickness reduction of material through uniform milling and corrosion activity were studied and compared using the high-frequency Rayleigh waves. Beat length effect due to continua slight shift difference between the two fundamental lamb wave modes, S_0_ and A_0_, were used to quantify thickness reduction. The technique was able to determine thickness reduction of about 0.1 mm; hence, it could quantify general corrosion impact or detect early corrosion activity, as a result implying the high sensitivity of the technique. In [119], thickness reduction of a thin copper plate was experimentally studied, compared with actual thickness reduction through milling. The variation of the plate thickness was monitored using group velocity. It was revealed that the two situations showed an agreeable result to the tune of 64% when the reduction was relatively small but deviated when it became more extensive. The study achieved a minimum detectable thickness reduction of 0.37% thickness. In [120], corrosion–erosion activity in plate and pipeline was studied using constant group velocity. The velocity of A_0_ mode was adopted in the study. The minimum detectable wall thickness reduction in the study is 1% of the general wall. This result showed an improvement in the sensitivity of the transducer. In [73], Lamb wave group velocity responsiveness to corrosion activity on the aluminium plate was studied. This study was numerically studied alongside the finite element solutions to determine the most sensitive frequency to thickness variation. It was revealed that sensitivity of guided waves varies with wave frequency and at low frequency, the wave is insensitive to small thickness variation of the metal. Interestingly, the fundamental symmetric, S_0_ and asymmetric A_0_ modes were recorded as insensitive. Mode conversion of the shear horizontal lamb wave (SH0 and SH1) after interaction with corrosion activity is studied in [121]. It was revealed that the group velocity of SH1 mode varies proportionately with variation in the structural thickness while SH0 mode remains nearly constant and insensitive to the thickness variation.

## 3. Effect of Environmental Conditions on Guided Wave and Ultrasonic Parametric Features

**Figure 11:**
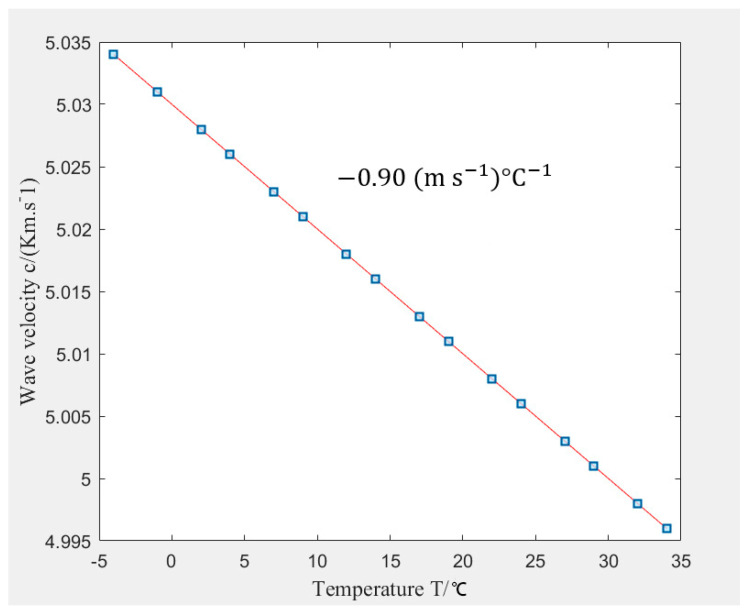
Temperature effect on Lamb wave velocity (R) [124].

Environmental factors vary uncontrollably and interact with installed asset structures. This interaction results in guided wave parameter variation, especially during thermal expansivity [122]. Since metallic structures are an excellent medium for propagating the inspecting wave and serve as host for the sensors, the effect of environmental variation on the metal could be transferred on the sensor directly or indirectly hence causing variation in the parametric features of the sensor signals. These variations mostly result in masking damage signals, thereby allowing failure to occur. Studies have been carried out to investigate the ambient temperature variation on the ultrasonic guided wave (host) and the transducers (actuator and sensor). In [123], Cawley made a comparison study of crack formation effect on the resonant frequency and impact of the beam’s length on the resonant frequency. The result showed that the change caused by 2% depth cut of the crack was 40 times smaller than that caused by a 2% increase in the beam’s length. The effect of temperature on UGW has been widely studied. In [124], Lui Zenghua et al. investigated the effect of temperature (−4 °C to 34 °C) on lamb wave, L (0,1) propagating on steel. The wave velocity was reported to vary inversely to increase in temperature, as shown in Figure 11. The retardation rate of the velocity was recorded to be 0.9 m/s for every degree of increase in temperature.

**Figure 12:**
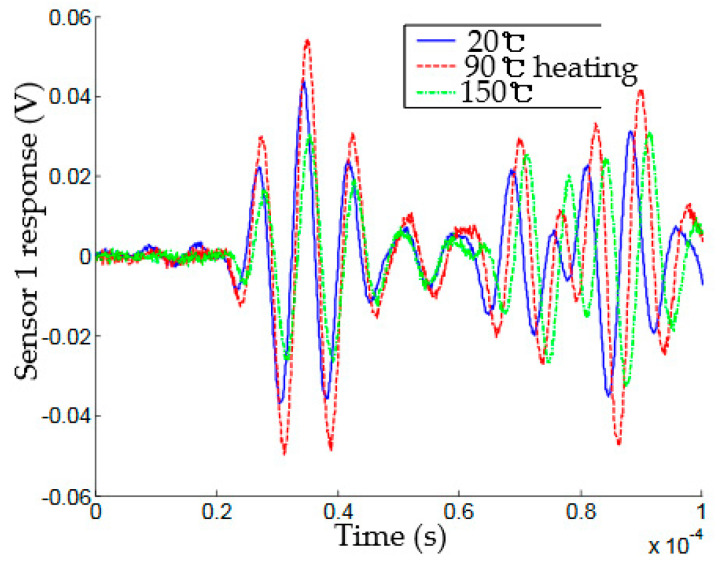
Temperature effect on PZT amplitude [126].

The study on anisotropic plate material carried out by Lanza et al. witnessed similar behaviour but on the wave signal amplitude [3]. It was observed that the wave amplitude increases at the temperature below 20 °C and decreases at a temperature above 20 °C. This effect is suggested to be attributed to the competing roles of the PZT coefficients and the dielectric permittivity. When the parameters were isolated, the amplitude response to temperature variation was observed to be proportional. Similar proportionality response was observed when the high-temperature transducer array coupled in an empty pipeline was subjected to a temperature of 7–150 °C [125]. In the pitch-catch study of varying temperature(20–150 °C) effect on guided wave performed by Raghavan and Cesnik in [126], the response was observed to be nonmonotonic in the measured amplitude as shown in Figure 12. The amplitude response signal increased when the temperature is 20–90 °C but decreased when it is 90–150 °C. This behaviour was subjected to further theoretical and experimental studies. Its behaviour was attributed to the assumption of perfect bonding and constant adhesive layer properties through the temperature range.

Temperature influence on the propagating wave time shift ${\left[\frac{\partial t}{\partial \sigma }\right]}_{T}$ and group velocity, $\nu_{g}\left(T,f\right)$ were modelled in Equations (18) and (19) respectively [127].

(18) $${\left[\frac{\partial t}{\partial \sigma }\right]}_{T}=\frac{d}{\nu }\left[\frac{1}{E}+\frac{K}{\nu }\right]$$

(19) $$\nu_{g}\left(T,f\right)=\left[23\times \frac{1}{T}+4.37\right]-\left[\frac{f}{175}-0.29\right]$$

where $\nu_{g}$ is the group velocity, *T* is the varying temperature, f is the frequency of the wave, *E* is the material young modulus and *K* is the coefficient of change in phase velocity with stress, and ν is the propagation velocity. Since $\frac{K}{\nu }$ > $\frac{1}{E}$, it could be inferred from Equation (18) that thermal influence on the wave velocity is more responsible for signalling time shift than the material mechanical property at peak temperature. Equation (19) was proposed in [127] to represent the group wave velocity function of varying ambient temperature and the propagating wave’s excitation frequency. The study on the influence of temperature variation on a diffused ultrasonic wave in a plate revealed waveform shift is an attribute of temperature. In contrast, distortion in waveform shape is proportional to the size of the flaw and perhaps its orientation [4]. In [128], the experimental study on steel thickness measurement at varying temperature (>21 °C) revealed an inverse relationship between temperature and the velocity of the ultrasonic wave. Based on the analysis, it was suggested that material young modulus E is the only property that could significantly contribute to variation in ultrasonic velocity under varying temperature and stress conditions as in Equation (20).

(20) $$C_{l}={\left[\frac{E.g}{\rho }.\frac{1-v}{\left(1+v\right)\left(1-2v\right)}\right]}^{\frac{1}{2}}$$

where *E* is the material young modulus, v is the Poisson ratio of the material, g is the acceleration due to gravity, and ρ is the density of the material.

**Figure 13:**
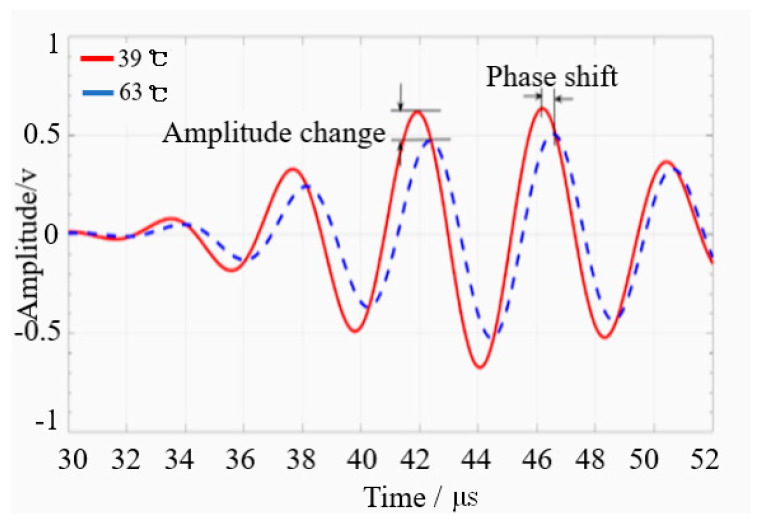
Typical signal differences under different temperatures [5].

Some other research studies on temperature influence on a guided wave and wave inspection parameters can be read in [6,7,8,9,10,129,130,131]. Figure 13 showed the significant effect of temperature variation that resulted in amplitude distortion and signal phase shift [5].

In [132], the influence of temperature variation (0–50 °C) on the ultrasonic feature of pulse-echo between motor oil and 1018 low carbon steel was studied. The attenuation of oil was concluded to affect the wave absorption coefficient significantly. This implies that motor oil is not a suitable interface coupling medium for GWUT under an elevated temperature, although it could limit corrosion development at the interface.

Aluminium metals are predominantly used as a thin plate to construct more extensive parts of an aircraft. Its exposure to harsh and varying environmental conditions are suggested to contribute to its deterioration and possible failures. In [67], temperature variation (−20–60 °C) effect on PZT propagating wave on an aluminium plate was experimentally studied. The wave amplitude and velocity were revealed to be inversely proportional to temperature variation. Towards 60 °C, the amplitude variation was about 30% while it was 2.5% on the velocity. This observation suggested that the tendency of missing damage signal at an elevated temperature of the structure is high.

Although it has been shown that temperature variation significantly affects the guided wave mechanical properties and ultrasonic wave parametric features, most of the studies were performed on pristine materials under controllable temperature with a marked range [67,91,128,132,133,134].

The unified effect of temperature con other environmental factors on sensor parametric features has not been given sincere attention than the investigative study done on temperature effect alone.

Conversely, the temperature effect on the PZT ultrasonic sensor’s parametric feature has been applied in a very innovative way to monitor an elevated temperature in a heated spiral spring [135]. The ToF of the pulse-echo signal after interaction with the structural predefined embodiment reflectors formed the basis for distributed temperature measurement. The comparable results showed that the ultrasonic waveguide measurement and the temperature measurements are acceptable with a maximum mean error of about 1–3 °C. The novel techniques could be deployed to measure assets’ temperature in an economical approach since a sensor could monitor the asset’s distributed temperature.

Compensating for the environmental effects on wave information signal is an approach to mitigate temperature and improve the positive probability of flaw detection in the structure. Different techniques have been applied to this effect, such as optimal baseline selection, baseline signal stretch technique, linear discriminant analysis [26,136,137]. As earlier mentioned, variation in temperature results in ultrasonic phase shift or delay in the signal arrival, reducing the sensitivity, and probability of damage detection. In the absence of temperature, determining the damage and its location would be a simple statistical evaluation between the baseline waveform signal and the current monitored signal. In [26], combined optimal baseline selection (OBS) and baseline signal stretch (BSS) techniques were used to suppress and compensate for the distorted information signal due to temperature variation. The results revealed that a combination of OBS and BSS performed better than simple subtraction method and OBS method. The compensation techniques have temperature range (Table 4) beyond which they are unreliable. Also, the size of the temperature reference signal interval attributes the ease of calculation process and the reference data set’s size.

In [136], the effect of temperature and flow rate of fluid in working pipeline acquired using principal component analysis techniques and Fourier transform, (FT) were used to classify the damage situation of the pipeline through linear discriminant analysis (LDA). The approach achieved an average of 98% damage detection accuracy. In [137], a modified baseline signal stretch (MBSS) method was used to enhance the damage detection of complex signals due to large temperature variation effect with fewer baseline data set. The improvement was on selecting and applying a range of stretch factors instead of using all at a given time, thereby retaining a minimum value of the residual envelope. It was validated experimentally on a plate structure. The study revealed that MBSS could detect damage in a structure at a higher temperature variation, hence it has more comprehensive application than BSS, as shown in Table 5.

In [138], dynamic time warping temperature compensation was studied to map the monitored data set with the baseline data set. The technique performed better than the stretch-based method at high-temperature variation. The obtained correlation coefficients at different study scenarios were steadily above 0.75, unlike 0.35–0.45 obtained in the stretch-based method. To ensure that the weak damage signature is not hidden in the noise, a statistical step detector approach was used to quantify the damage as correlation coefficients reduce over time. However, the technique’s computational cost is high, requiring any other method to reduce the cost while ensuring accuracy in damage detection. The flexibility of the dynamic warping technique is suspected to be unreliable as it has not been subjected to many case scenarios.

In [139], the structural health of material was studied by monitoring the variation of the propagating frequencies of the exciting wave using PZT transducers. The Gaussian kernel was used in support vector machine (SVM) to determine the damage states similarities at each measured frequency. The method showed a 90% performance score although, environmental factors were not included in the study or considered. Hence, factoring environmental conditions effect into the approach may improve its performance score above 90%.

## 4. Impedance-Based Model Damage Detection

**Figure 14:**
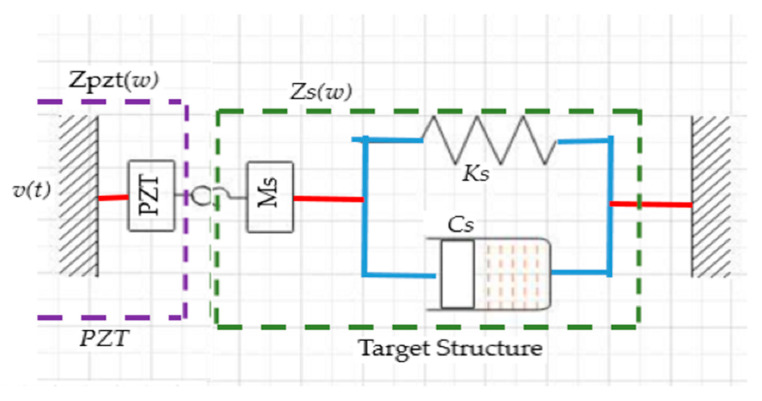
1-D PZT–target structure interface [143].

It is an acknowledged fact that temperature variation brings about changes in the PZT constant properties, adhesive layer properties between the PZT and the target structure, mechanical properties of the target structure such as young modulus [4,140,141]. Principally, the PZT is bonded to the target structure using an adhesive agent. When an appropriate excitation voltage is applied on the PZT, it would exert acoustic pressure via adhesive layer to the target structure and cause its particles to vibrate in their mean positions. The interaction of the PZT sensor with the target structure in SHM has been modelled in different ways. The essence is to understand their behaviour when there is an existence of damage in the target structure. Among these models are the static and the dynamic finite element models that used the static properties of PZT and structural stiffness to determine the output characteristics of the PZT sensor. The impedance-based-model coupled electro-mechanical system has been more interesting and advantageous than the other two models [142,143]. However, these models are of one degree of freedom (1-DOF) because they considered only the interface between the PZT and the host structure. The physical model of the system is shown in Figure 14 and its simplified model equation for impedance coupling, Z(ω) the PZT and the target structure are given in Equation (21) while Equation’s structural impedance is Equation (22). For brevity, the equation terms are defined in Table 6.

(21) Z(ω)={iωwpztlpzttpzt[ε^33T−1Zpzt(ω)Zs(ω)+1d312Y^31E]}−1

(22) $$Z_{s}\left(\omega \right)=m\frac{\omega^{2}-\omega_{n}^{2}}{\omega }+C_{s}$$

Equations (21) and (22) showed that impedance-based monitoring of structural damage is a function of the frequency band that must be selected appropriately to detect damage change in a structure effectively. In the case of excitation frequency of the PZT matches the natural frequency of the target structure, the structural, mechanical impedance could be compared with the mechanical impedance of the PZT thereby resulting in an electro-mechanical impedance given in Equation (23).

(23) Z(ω)={iωwpztlpzttpzt[ε^33T−1Zpzt(ω)Cs+1d312Y^31E]}−1

**Figure 15:**
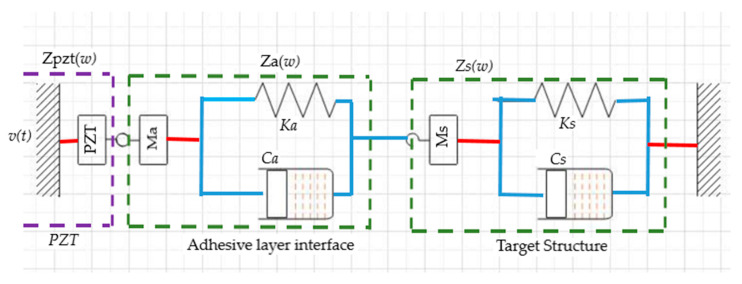
Modified PZT–host structure interface [142].

Hence, damage detection in a structure could be detected through the quantification of the measured impedance variation. However, when PZT is coupled on the target structure without an adhesive agent, it results in a high impedance mismatch due to air that serves the adhesive agent’s intended function. Over time, the adhesive layer becomes weak and debonds from the interface, allowing air to exist in between the PZT and the target structure interface. Exposure to high environmental temperatures promotes this effect. This effect degrades the SHM system’s sensitivity and makes damage detection difficulty. Hence, there is a need to factor in the impedance-based model the adhesive layer’s influence. In furtherance of EMI based 1-D model, an improvement was made on the model to accommodate the adhesive layer [142]. The modified model of the system is in Figure 15. The resulting 2-DOF EM impedance between PZT-interface-host structure is given in Equation (24).

(24) Z(ω)={iωwpztlpzttpzt[ε^33T−1Zpzt(ω)Z^(ω)+1d312Y^31E]}−1

where

(25) $$\hat{Z}\left(\omega \right)=\frac{K_{11}K_{22}-{\left(K_{12}\right)}^{2}}{i\omega K_{22}}$$

In this model, the output characteristics of PZT is determined via its dynamic properties and structural impedance. By varying the stiffness of the model depicts changes in the target structure.

The impedance-based model was used by Bin Lin et al., in [7] to study the durability and survivability of PWAS. The impedance of one free bond PWAS and one bonded PWAS on metal were measured after been subjected to different situations of harsh and mild temperature for a shorter time. The situations were captured as cryogenic and high temperature, temperature cycling, freeze–thaw, outdoor environment, operational fluids, large strains, and fatigue load cycling [144]. It was concluded that PWAS could function well in cryogenic temperature but not in high temperature. It could be said that the durability and survivability of PZT are subject to the nature of the working environment and condition of the system operation. In [145], the durability of PZT actuators under the influence of electro-thermo-mechanical condition was studied.

Although PZT could be perfectly bonded to the target structure’s surface, the adhesive agent could weaken after a prolonged time in service. At this point, the performance of the PZT will start degrading, and integrity of the SHM compromised. PZT actuator unfortunate damage could occur before or during structural health monitoring. Its failure during operation could impair structural damage detection by generating signals that interfere with structural damage signal [146,147]. This effect brings complexity in damage signal interpretation. It is vital to determine when a sensor has failed and isolate it from the network of others. Since a failed sensor signal can interfere with the structural damage signal, using the real admittance to discriminate the failed sensor damage signal from that of the host structure, electro-mechanical impedance (EMI) technique has shown strength in this regard [144,146,147]. Sensor self-diagnosing was studied in [10] using electrical impedance and in [147] using real admittance of the EMI-based model. In [147], the authors used the resonant frequency shift of the real admittance caused by structural impedance and root mean square deviation (RMSD) index to achieve this discrimination. However, the study did not consider the effect of temperature variation.

## 5. Conclusions

A review is presented on damage inspection, severity, and temperature influence on the inspecting guided wave and its parametric features. The sensor’s choice for asset integrity monitoring depends primarily on the structure’s nature and the targeted flaw. Different ultrasonic parametric features are used in determining the existence of flaw or damage in the structure. However, it is pertinent that it must be highly responsive to the intended flaw, otherwise a high rate false detection alarms is bound to occur and reliability of the structure would be jeopardized.

In contrast with the conventional ultrasonic inspection technique, an inspection of a large structure area from a test point is possible with GWUT. This improvement brings about a reduction in the cost of structural assessment and the amount of time required to inspect its health state. Although at the expense of ease of interpretation, the ability of guided wave ultrasonic technique to generate multi-mode waves is a good characteristic that aids in monitoring and detecting sundry damage in the structure.

From the DI reviewed works, it was observed that damage indices in time-domain are mostly used to monitor the existence of macroscale damage in structures. These are cracks and other damage that have developed and could significantly reflect or scatter the propagating wave. The microscale damage under breathing crack mechanism results in higher harmonics formation and makes signal interpretation difficult in time-domains. In such a situation, frequency-domain DI is mostly adopted to diagnose and interpret its existence.

Additionally, DI’s effectiveness is a function of its responsiveness to the presence and variation of damage while being robust to unwanted interfering signals. However, most of the IDs depend mainly on pristine structure values to be reasonably accurate, which is difficult to obtain from the existing asset that are many years old. All the DIs are mono damage identifier or mono severity indicator and unfit to define much damage at a time.

The influence of temperature in damage detection and localization is still a challenge. However, different temperature compensation techniques exist but at the expense of high computational cost and reference to pristine values which are not readily available. The effect of varying temperature on waveforms results in a time shift of the inspecting signal with a high probability of missing flaw detection at an elevated temperature. The influence could worsen at high temperature and large defect size leading to system unreliability. Thermal conductivity is perceived as a key factor in ultrasonic attenuation under temperature variation and the ultrasonic velocity’s proportionality behaviour with temperature. Temperature variation is the only environmental factor considered in most of the studies, whereas the monitoring system could be affected by other environmental factors when deployed to the field.

Study on the effect of crack or breathing crack in a corroded area on GWUT parameters under varying temperature influences is yet to be studied. Situations are abounding where corrosion activities may cover cracks, and structure diagnosis would reveal the existence of corrosion. This could cause misinformed asset reliability and unscheduled system failure. Also, damage detection in the dynamic stressed thin plate under the influence of thermomechanical condition has not been explored using GWUT. Most metallic structures are subjected to dynamic stress and environmental conditions while in service. These two conditions are detrimental to damage detection in structures using GWUT. Leveraging the potentialities of GWUT for structure health inspection, determining the effect of these combined conditions is significant in deciding damage severity, location, and orientation in a structure. Understanding the dynamics of these aforementioned challenges with respect to ultrasonic guided waves will open a new research discussion. The solution will increase the effectiveness of GWUT in achieving informed and reliable damage detection in metallic assets.

## Figures and Tables

**Table 1:** Parameter definition.

Electrical Parameters	Mechanical Parameters	Constants and Couplants
$E_{j}$, applied electric potential	$T_{j}$, material tensor stress	$e_{jj}$, third-rank tensor
$D_{i}$, particle displacements	$S_{i}$, material tensor strain	$\epsilon_{ij}^{s}$, permittivity for constant strain
		$C_{ji}^{E}$, elastic stiffness tensor for constant electric field

**Table 2:** Transducer categories concerning spectrum response.

S/N	Transducer Series	Remarks of Merit
1	High resolution (HR) Series	Heavily damped Most broadband Effective for structure thickness gaging
2	General purpose(GP) Series	Less damped Bandwidth is between the narrow banded and broadband series Effective in detecting the majority of a structural flaw
3	High gain (HG) series	Less damped and tuned Most narrow banded Effective in detecting flaw on highly attenuated, rough surface or relatively thick materials.

**Table 4:** Temperature range and interval of some existing compensation technique.

Compensation Method	Temperature Range	Maximum Compensation Interval	Reference Signal Interval
BSS	5–40 °C	5 °C	0.1 °C
OBS	22–32 °C	10 °C	0.1 °C
BSS + OBS	21.5–31.5 °C	10 °C	0.5 °C

**Table 5:** Summary of result indicating the largest temperature change for which the damage could be accurately localized by applying temperature correction methods.

Name	Aluminium Small Artificial Damage	Aluminium Crack	CFRP BVID
BSS	Up to ΔT = 3 °C	Up to ΔT = 15 °C	Up to ΔT = 8 °C
MBSS	Up to ΔT = 13 °C	Up to ΔT = 23 °C	Up to ΔT = 28 °C

**Table 6:** Equation terms definition.

Terms	Definition
${\hat{Y}}_{31}^{E}$	Complex young modulus of the PZT at zero electric field
$d_{31}^{2}$	PZT coupling constant in 1-direction
${\hat{\varepsilon }}_{33}^{T}$	Complex dielectric at zero electric field
$Z_{pzt}\left(\omega \right)$	Electrical impedance of the PZT
$Z_{s}\left(\omega \right)$	Structural mechanical impedance of the target structure
$w_{pzt}$	Width of the PZT
$l_{pzt}$	Length of the PZT
$t_{pzt}$	Thickness of the PZT
$C_{s}$	Damping coefficient of the structure
ω	Angular frequency of the excitation voltage applied on the PZT
*m*	Mass of the target structure
$\omega_{n}$	The angular natural frequency of the target structure
$K_{ij}$	Dynamic stiffness of the 2-DOF
$\hat{Z}\left(\omega \right)$	Structural mechanical impedance of the interface-target structure

## Data Availability

The data presented in this study are available on request from the corresponding author. The data are not publicly available because it forms part of an ongoing study.
